# Are patients in heart failure trials representative of primary care populations? A systematic review

**DOI:** 10.3399/bjgpopen18X101337

**Published:** 2018-04-24

**Authors:** Nicholas D Gollop, John Ford, Pieter Mackeith, Caroline Thurlow, Rachel Wakelin, Nicholas Steel, Robert Fleetcroft

**Affiliations:** 1 MRC Clinical Research Fellow in Cardiology, Norwich Medical School, University of East Anglia, Norwich, UK; 2 NIHR Clinical Research Fellow, Norwich Medical School, University of East Anglia, Norwich, UK; 3 Academic Clinical Fellow in Primary Care, Norwich Medical School, University of East Anglia, Norwich, UK; 4 Academic Clinical Fellow in Primary Care, Norwich Medical School, University of East Anglia, Norwich, UK; 5 Academic Clinical Fellow in Primary Care, Norwich Medical School, University of East Anglia, Norwich, UK; 6 Professor in Public Health, Norwich Medical School, University of East Anglia, Norwich, UK; 7 Honorary Senior Fellow, Norwich Medical School, University of East Anglia, Norwich, UK

**Keywords:** heart failure, primary health care, drug treatment, guidelines

## Abstract

**Background:**

Guidelines recommend drug treatment for patients with heart failure with a reduced ejection fraction (HFrEF), however the evidence for benefit in patients with mild disease, such as most in primary care, is uncertain. Importantly, drugs commonly used in heart failure account for one in seven of emergency admissions for adverse drug reactions.

**Aim:**

To determine to what extent patients included in studies of heart failure treatment with beta-blockers, angiotensin-converting enzyme (ACE) inhibitors, and aldosterone antagonists were representative of a typical primary care population with HFrEF in England.

**Design & setting:**

Systematic review of randomised controlled trials (RCTs) of drug treatment in patients with HFrEF.

**Method:**

MEDLINE, MEDLINE In-Process, EMBASE, and CENTRAL were searched from inception to March 2015. The characteristics of the patient’s New York Heart Association (NYHA) classification were compared with a primary care reference population with HFrEF.

**Results:**

Of the 30 studies included, two had incomplete data. None had a close match (defined as ≤10% deviation from reference study) for NYHA class I disease; 5/28 were a close match for NYHA class II; 5/28 for NYHA class III; and 18/28 for NYHA class IV. In general, pre-existing cardiovascular conditions, risk factors, and comorbidities were representative of the reference population.

**Conclusion:**

Patients recruited to studies typically had more severe heart failure than the reference primary care population. When evidence from sicker patients is generalised to less sick people, there is increased uncertainty about benefit and also a risk of harm from overtreatment. More evidence is needed on the effectiveness of treatment of heart failure in asymptomatic patients with NYHA class I.

## How this fits in

Heart failure is common in primary care and carries a high morbidity and mortality which is associated with the degree of failure; beta-blockers, ACE inhibitors or angiotensin II receptor blockers (ARBs), and aldosterone antagonists have all been shown to reduce mortality and morbidity, but also carry a significant risk of adverse drug reactions. This study shows that patients with heart failure in primary care tend to have mild heart failure, but the evidence for effectiveness for these drugs comes from a population with more severe heart failure. More evidence is needed for the effectiveness of these treatments in populations typical of primary care.

## Introduction

HFrEF is a common chronic, debilitating disease which has a prevalence of 0.7% and affects 400 000 adults in the UK.^1^ The annual cost of heart failure to the NHS is around 2% of its total budget, and approximately 70% of this total is due to the costs of hospitalisation.^2^ There is a large variation in clinical presentation of heart failure, with some patients having no symptoms at the time of diagnosis whereas others have significant morbidity. The diagnosis is made based on the presence of signs and symptoms of heart failure and through the use of echocardiography to measure left ventricular ejection fraction (LVEF).^3^ An LVEF <40% confirms a diagnosis of HFrEF, which has been extensively studied in the literature.

Symptoms of heart failure can be graded using the NYHA functional classification into one of four categories (Box 1).^4^ In one study of UK primary care patients with HFrEF, 47% had no symptoms (class I), 36% had mild symptoms (class II), 7% had moderate symptoms (class III), and 10% had severe symptoms (class IV).^5^ Mortality rates from heart failure are high; one UK cohort study reported that 14% (95% confidence interval [CI] = 11% to 18%) of patients died within 6 months of diagnosis.^6^ Patients with higher NYHA symptom scores have a worse prognosis, although even patients with mild heart failure have higher mortality than the general population.^7^

Several large trials have found a reduction in mortality and hospitalisation in patients with systolic heart failure following treatment with beta-blockers, ACE inhibitors, and aldosterone antagonists.^8,9^ These drugs have also been shown to be cost-effective for the treatment of heart failure.^10^ This evidence has led to guideline recommendations adopting these treatments for systolic heart failure across the world.^2,7,11,12^ The National Institute for Health and Care Excellence (NICE) heart failure guideline recommends that all primary care patients with systolic heart failure should be offered beta-blockers and ACE inhibitors, regardless of NYHA class. This indicator is supported by evidence generalised from higher risk populations (NYHA classes III–IV), in which there is clear evidence of benefit for beta-blockers and ACE inhibitors, but the evidence of benefit in lower risk populations is more equivocal.^13,14^

The applicability of guideline recommendations for management of diseases (including heart failure) in primary care has recently been questioned as this research is rarely conducted in representative populations.^13^ This question is important in heart failure because the effectiveness of treatment may depend on the severity of disease, and beta-blockers and ACE inhibitors carry significant morbidity risk, accounting for approximately one in seven emergency hospital admissions due to adverse drug reactions.^15^

The aim of this study was to determine to what extent patients included in studies of heart failure treatment with beta-blockers, ACE inhibitors, and aldosterone antagonists were representative of the NYHA class and other characteristics of a typical primary care population with heart failure in England.

## Method

A literature search was undertaken to identify RCTs of systolic heart failure drugs. MEDLINE, MEDLINE In-Process, EMBASE, and CENTRAL were searched from inception to March 2015. The search strategy for MEDLINE (further information available from the authors on request) was modified for other databases. Titles and abstracts were screened by two authors independently, according to the following pre-specified inclusion and exclusion criteria.

Inclusion criteria were RCTs which included patients with HFrEF. Intervention drugs included ACE inhibitors, beta-blockers, ARBs, and aldosterone antagonists, such as spironolactone and eplenerone. There were no language restrictions. Exclusion criteria were studies with a follow-up of <6 weeks duration, those comprising a single-dose regimen, and studies not judged to be generalisable to a primary care population (such as one study of patients on dialysis). Disagreements were resolved through discussion or by a third researcher, and full text articles were retrieved for each abstract meeting the inclusion criteria.

Data were extracted from each included study into a template which included study design, intervention, inclusion and exclusion criteria, baseline characteristics, primary outcome, and mortality data. Data extraction was checked by a second researcher and any disagreements were resolved through discussion or by a third researcher. Authors were contacted for individual-level data. No authors shared individual-level data and the difficulties accessing these data have been described elsewhere.^16^ Study exclusion was guided by pre-defined exclusion criteria as described.

Data was used from the largest study on the prevalence of heart failure in the UK, that is the Echocardiographic Heart Study of England Screening (EHES) study.^5^ This study randomly selected a large population of 6286 people aged ≥45 years and, of the five studies of heart failure prevalence identified, was the best fit to an English population.^17–19^ The EHES study had a high participation rate of 63% (3960 patients) and wide geographical spread of populations which was representative of inner-city, urban, suburban, and rural communities. The EHES study was used as the reference population throughout this study.

For each study, the NYHA class, baseline cardiovascular risk factors, baseline cardiovascular comorbidities, and use of heart failure drugs were analysed. These outcomes were compared between the reference study and each extracted study. Each patient-specific variable was compared to the reference study in terms of prevalence or frequency of use. To allow quantification of similarity between the selected study population and the reference study population, the percentage deviation was assessed and allocated as being a close match, fair match, or poor match. If the extracted study population had a ≤10% deviation from the reference study, it was termed as a close match; if the deviation was 11–20%, it was termed a fair match; and if the deviation was >20%, it was termed a poor match These parameters were set out *a priori*. For example, if a study reported 10% class I, 25% class II, 40% class III, and 25% class IV, to assess close match a 10% absolute deviation was applied (that is, 0–20, 15–35, 30–50, and 15–35% respectively) and compared it to classes in the reference population (47, 36, 7, and 10% respectively). This worked example is shown in Table 1 (further information available from the authors on request).

**Box 1.** New York Heart Association classes of heart failure.^4^

Class	Patient symptoms
I	No limitation of physical activity. Ordinary physical activity does not cause undue fatigue, palpitation, dyspnoea (shortness of breath).
II	Slight limitation of physical activity. Comfortable at rest. Ordinary physical activity results in fatigue, palpitation, dyspnoea (shortness of breath).
III	Marked limitation of physical activity. Comfortable at rest. Less than ordinary activity causes fatigue, palpitation, or dyspnoea.
IV	Unable to carry on any physical activity without discomfort. Symptoms of heart failure at rest. If any physical activity is undertaken, discomfort increases.

**Table 1:** Example assessment of an extracted paper compared to the reference population

	Class I	Class II	Class III	Class IV
Reference population, %	47	36	7	10
Extracted study, %	10	25	40	25
Extracted study with 10% deviation, %	0–20	15–35	30–50	15–35
Closeness of match, %	>20	11–20	>20	11–20
Closeness of match, label	Poor	Fair	Poor	Fair

## Results

**Figure 1.**
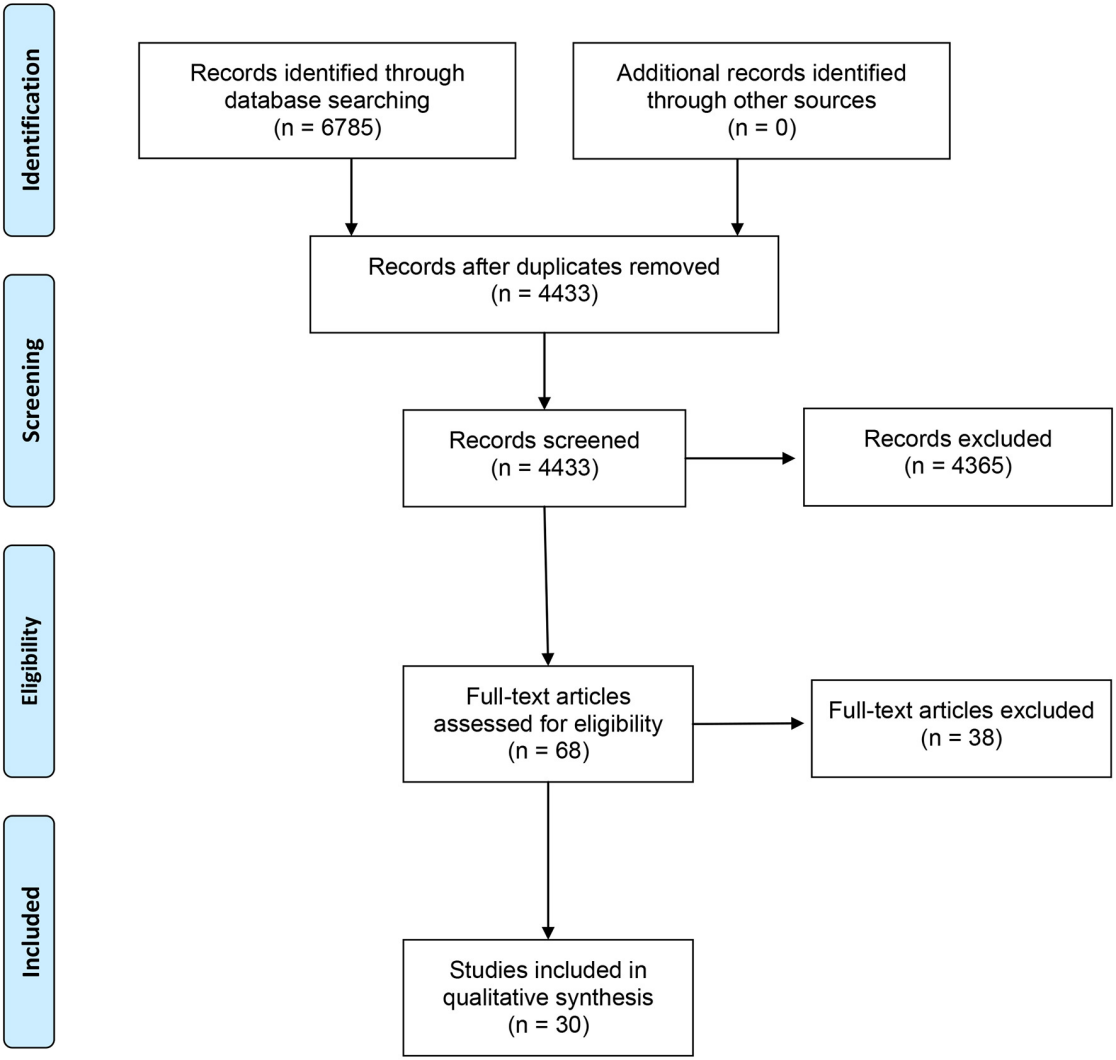
PRISMA diagram.

Literature searching identified 6785 studies, 4433 after de-duplication (Figure 1). Thirty RCTs met the inclusion criteria, representing 43 454 patients with HFrEF. Characteristics of included studies are shown in **Table 2**. Of the included studies, 13 investigated beta-blockers, 8 ACE inhibitors, 6 ARBs, and 4 spironolactone. One study compared ACE inhibitors and ARBs (ELITE I, 2000). In the 30 extrapolated studies, sample size ranged from 59–5010 participants. Follow-up ranged from 3–73 months.

**Table 2:** Characteristics of included studies

Study ID	Comparison	Number of participants	Primary outcome	Follow-up, months
AREA-CHF 2009^20^	Canrenone Placebo	231 236	Change in LV diastolic volume	12
BEST 2003^21^	Bucindolol Placebo	114 112	Death and heart failure hospitalisation composite	19
Borghi 2013^22^	Ramipril Zofenopril	73 102	Survival	73±14
CARNEBI 2013^23^	Carvedilol Bisoprolol Nebivolol	61 crossover	NYHA class, biochemistry, and physiological testing	6 (2 x 3 crossover)
CELICARD 2000^24^	Celiprolol Placebo	62 62	Functional score — Goldman score	12
CHARM Added 2003^25^	Candesartan Placebo	1011 1014	Cardiovascular death or unplanned hospital admissions for worsening CHF	34
CHARM Alternative 2003^26^	Candesartan Placebo	1273 1271	Cardiovascular death or unplanned hospital admissions for worsening CHF	41
CIBIS 1994^27^	Bisoprolol Placebo	320 321	All-cause mortality	23
CIBIS 1999^28^	Bisoprolol Placebo	1327 1320	All-cause mortality	16
Cicoira 2002^29^	Spironolactone Placebo	54 52	Physiological or functional improvement	12
Cohn 2001^30^	Valsartan Placebo	2511 2499	All-cause mortality, and combined mortality and morbidity	23
Colucci 1996^31^	Carvedilol Placebo	232 134	Disease progression and death composite	12
COMET 2003^32^	Carvedilol Metoprolol	1511 1518	All-cause mortality	58
Dalla-Volta 1999^33^	Delapril Enalapril	88 91	Physiological or functional improvement	12
ELITE II 2000^34^	Losartan Captopril	1578 1574	All-cause mortality	18
Kum 2008^35^	Add on Irbesartan Placebo	50 50	6MHW, Minnesota (QoL), peak exercise capacity on treadmill	12
Liu 2014^36^	Metoprolol Conventional therapy	77 77	NYHA class, LVESD, LVEDD, LVEF, 6-min walking distance, medication safety	6
MAIN CHF II 2014^37^	Bisoprolol Carvedilol	21 14	Clinical and functional status, mortality rate	8
MERIT-HF 1999^48^	Metoprolol CR Placebo	1990 2001	All-cause mortality	12
Munich 1991^38^	Captopril Placebo	83 87	Cardiovascular-cause mortality	33
Pitt 1999^9^	Spironolactone Placebo	822 841	All-cause mortality	24
Riegger 1999^39^	Candesartan 4 mg Candesartan 8 mg Candesartan 16 mg Placebo	211 208 212 213	Increase in exercise tolerance, reduction in NYHA class	3
SENIORS 2005^40^	Nevovitol Placebo	1067 1061	All-cause mortality and time to first CVD admission	21
SOLVD 1991^41^	Enalapril Placebo	1285 1284	Clinical and functional status, mortality rate	41.4
SOLVD 1992^42^	Enalapril Placebo	2111 2117	Clinical and functional status, mortality rate	37.4
Sturm 2000^43^	Atenolol Placebo	51 49	Worsening heart failure or death	24
US Carvedilol 2001^44^	Carvedilol Placebo	Black: 127 Not Black: 569 Black: 90 Not Black: 308	Ethnicity (self-reported), ejection fraction, clinical status, and major clinical events	15
Yodfat 1991^45^	Captopril Placebo	41 43	Functional status	3
Zannad 1998^46^	Fosinopril Placebo	122 132	Cardiovascular mortality and event-free survival	12
Zannad 2011^47^	Eplenerone Placebo	1364 1373	Cardiovascular mortality and event-free survival	21

6MHW = 6-minute hall walk. CHF = congestive heart failure. CVD = cardiovascular disease. LV = left ventricular. LVEDD = left ventricular end-diastolic diameter. LVEF = left ventricular ejection fraction. LVESD = left ventricular end-systolic diameter. NYHA = New York Heart Association. QOL = quality of life.

Characteristics of the reference population are shown in Table 3. The overall mean age was 69 years, and 81% of the reference population was male. Most patients had NYHA class I (47%) and only 17% of patients had class III or IV.

**Table 3:** Summary of the ejection fraction <40% cohort for the reference population

Characteristic	Total (*n* = 72), *n* (%)
Mean age, years (SD)	69 (9)
Female	14 (19)
Male	58 (81)
Ever smoked	50 (69)
Non-white	2 (3)
Any electrocardiogram abnormality	2 (3)
Mean height, metres (SD)	1.71 (0.09)
Mean weight, kg (SD)	80.8 (14.6)
Mean heart rate, beats per min (SD)	77.3 (17.8)
Mean forced expiratory volume at 1 second, litres (SD)	2.11 (0.76)
Mean forced vital capacity, litres (SD)	2.55 (0.85)
Mean systolic blood pressure, mmHg (SD)	148.4 (21.1)
Mean diastolic blood pressure, mmHg (SD)	87.1 (12.3)
**New York Heart Association class**	
I	34 (47)
II	26 (36)
III	5 (7)
IV	7 (10)
**History**	
Myocardial ischaemia	38 (53)
Angina	26 (36)
Hypertension	28 (39)
Diabetes	11 (15)
Family myocardial ischaemia (age <65 years)	25 (35)
**Medication taken**	
ACE inhibitors	19 (26)
Diuretics	26 (36)
Beta-blockers	9 (13)
Calcium antagonists	15 (21)
Aspirin	38 (53)
Digoxin	5 (7)

SD = standard deviation.

### NYHA class

Table 4 shows heart failure RCTs compared to the reference population, stratified by NYHA class. Of the 30 studies, 28 had complete data on NYHA classes. None of the studies had a close match for NYHA class I disease, 3/28 (11%) displayed a fair match, and 25/28 (89%) a poor match. For NYHA class II 5/28 (18%) studies has a close match, 9/28 (32%) a fair match, and 14/28 (50%) a poor match. For NYHA class III, 5/28 (18%) displayed a close match, 3/28 (11%) a fair match, and 20/28 (71%) a poor match. For NYHA class IV, 3/28 (11%) displayed a fair match, and 18/28 (64%) displayed a close match and 7/28 (25%) had a poor match.

**Table 4.** NYHA classification in heart failure RCTs compared to the reference population.

		**NYHA class^5^**			
Heart failure RCTs	*N*	I^a^, %	II^b^, %	III^c^, %	IV^d^, %
SOLVD 1992	4228	11–20	<10	<10	<10
Munich 1991	170	11–20	11–20	11–20	<10
Borghi 2013	175	11–20	11–20	11–20	<10
US Carvedilol 1996	1094	>20	<10	>20	<10
Liu 2014	154	>20	<10	>20	<10
CHARM Added 2003	2548	>20	<10	>20	<10
MERIT-HF 1999	3991	>20	<10	>20	<10
Zannad 1998	254	>20	>20	<10	<10
CELICARD 2000	124	>20	11–20	>20	<10
CHARM Alternative 2003	2028	>20	11–20	>20	<10
SENIORS 2005	2128	>20	11–20	>20	<10
SOLVD 1991	2569	>20	11–20	>20	<10
COMET 2003	3029	>20	11–20	>20	<10
Cicoira 2002	106	^e^	^e^	^e^	^e^
CARNEBI 2013	183	>20	>20	<10	>20
MAIN CHF II 2014	59	>20	>20	<10	>20
Colucci 1996	366	>20	>20	<10	>20
Zannad 2011	2737	>20	>20	>20	<10
Sturm 2000	100	>20	>20	>20	<10
Cohn 2001	5010	>20	>20	>20	<10
CIBIS 1994	641	>20	>20	>20	<10
CIBIS 1999	2647	>20	>20	>20	<10
ELITE II 2000	3152	>20	11–20	>20	>20
Kum 2008	100	>20	11–20	>20	>20
Rieger 1999	844	>20	>20	11–20	>20
BEST 2003	226	>20	>20	>20	>20
Dalla-Volta 1999	179	>20	>20	>20	>20
AREA-CHF 2009	382	>20	>20	>20	>20
Pitt 1999	1663	>20	>20	>20	>20
Yodfat 1991	84	^e^	^e^	^e^	^e^

^a^47% of reference population. ^b^36% of reference poulation. ^c^7% of reference population. ^d^10% of reference population. ^e^Insufficient information to calculate deviation. RCT = randomised controlled trial. NYHA = New York Heart Association.

### Baseline cardiovascular risk factors

Cardiovascular risk factors were largely representative of the reference population (further information available from the authors on request). Of the 30 studies, 25 (83%) had a close match to the age of the reference population, which was a mean of 69 years. Nineteen studies (63%), had a close match with the sex characteristics of the reference population, which was 81% male. The majority of extracted studies (20/30, 67%), did not present ethnicity data. Of those that did, 7/10 (70%) had a close match and 3/10 (30%) had a poor match with the reference population, which was 97% white. Of the 30 extracted studies, 23 (77%) did not present smoking status data. Of the seven that did, one (14%) had a fair match and six (86%) a poor match to the reference population, 69% of whom were smokers. A family history of premature myocardial infarction was not reported in any of the studies.

### Baseline cardiovascular comorbidities

The majority of the studies (23/30, 77%), reported the presence of pre-existing angina but 13/30 (43%) studies did not report the presence of previous myocardial infarction, pre-existing hypertension, or diabetes mellitus (further information available from the authors on request). In general, pre-existing cardiovascular conditions recorded in the extracted studies were representative of the reference population. When comparing for the presence of pre-existing myocardial infarction, 10/17 (59%) of the extracted studies had a close match, 6/17 (35%) had a fair match, and 1/17 (6%) had a poor match to the reference population, which reported a prevalence of 53%. A similar trend was noted for hypertension, for which 7/17 (41%) of the extracted studies had a close match, 3/17 (18%) had a fair match, and 7/17 (41%) had a poor match to the reference population, which had a reported prevalence of 39%.

For diabetes mellitus, 7/17 (41%) of the extracted studies had a close match, 6/17 (35%) had a fair match, and 4/17 (24%) had a poor match to the reference population (reported prevalence, 15%). As mentioned, the presence of angina was recorded in only seven studies. Of these, 3/7 (43%) had a close match, 3/7 (43%) had a fair match, and 1/7 (14%) had a poor match to the reference population (reference population reported prevalence, 36%).

### Use of heart failure drugs

The use of important heart failure drugs varied significantly across the analysed studies (further information available from the authors on request). Of the 30 studies, 20 (67%) did not report data on the use of aspirin. Of the remainder, 5/10 (50%) had a close match, 4/10 (40%) a fair match, and 1/10 (10%) had a poor match to the reference population, of whom 53% took regular aspirin.

Of the 30 extracted studies, 22 (73%) did not report data on the use of calcium channel blockers (CCBs). Of the remaining eight, four (50%) had a close match, and four (50%) had a fair match to the reference population, which reported CCB usage in 21%.

A large proportion of the extracted studies investigated beta-blockers and ACE inhibitors directly, and therefore were not assessed for prevalence of use of these therapies compared to the reference population. Of the 18 studies which did not study beta-blockers, 11 (61%) did report data on the proportion of patients using beta-blockers, and only three (27%) of these 11 had a close match to the reference population, which reported a frequency of 13%.

Of the 22 studies that did not directly study ACE inhibitors, eight (36%) did not report prevalence of use. Therefore only 14 (47%) of the 30 total extracted studies could be assessed for ACE inhibitors, all of which had a poor match to the reference population, which reported a frequency of 26%.

Eleven (37%) studies did not report data on the proportion of patients using digoxin. Of the remaining 19, two (11%) had a close match, two (11%) had a fair match, and 15 (79%) had a poor match to the reference population, which had a reported frequency of 7%.

Spironolactone and eplenerone were the study drug in 4/30 studies and these were therefore not assessed for similarity to the reference population. Of the remaining 26 studies that did not directly investigate these agents, 10 (38%) did not report prevalence of use data. As such, only 16/30 (53%) studies could be assessed for spironolactone and eplenerone use, all of which had a poor match to the reference population, which had a reported frequency of 36%.

The authors of this study examined the six studies that were a close match for NYHA class II participants for evidence of benefit for this class. Only one study (MERIT-HF) reported outcomes by NYHA class II,^48^ the remaining studies reported pooled outcomes for all NYHA classes. MERIT-HF reported no significant mortality reduction, but a reduction in two out of four secondary outcomes (development of congestive heart failure [CHF] and hospitalisations).

## Discussion

### Summary

Of the reference population representing a primary care population with HFrEF, 83% had mild symptoms in NYHA class I and II, however none of the 30 studies were matched closely with NYHA class I, and only 5/28 (18%) studies were a close match with NYHA class II symptoms. For patient characteristics of age, sex, ethnicity, previous myocardial infarction, hypertension, diabetes, and angina, >40% studies were closely matched to the reference population. For patient characteristics of smoking status; family history of premature heart disease; and the use of beta-blockers, ACE inhibitors, and the aldosterone antagonists spironolactone and eplenerone, <30% of studies were closely matched to the reference population. In this way, this systematic review has shown that these studies are not typically representative of the primary care population in England, with patients with more severe heart failure being overrepresented.

### Strengths and limitations

This study is the first systematic review to determine whether the types of patients included in studies of treatments for HFrEF were representative of a typical primary care population with HFrEF in England. A large study was used as the reference population,^5^ which randomly selected and screened the population for HFrEF, and the systematic review method of the present study was robust. While this reference study was published 15 years ago, and the characteristics of the primary care population and treatments have changed, it is closer to the time when the included RCTs were undertaken. The present authors had initially intended to obtain individual-level data for each NYHA class from each of the 30 identified studies, however, there were obstacles in terms of non-disclosure of further information from these studies' authors, who either failed to reply to repeated attempts to make contact or were unwilling for the present authors to access their trial data.^16^ There may be some overlap between classes, such as class I and II, which may have led to misclassification in either the reference study or the included trials. Only trials which recruited patients with heart failure were included, and there is a possibility that some trials with a subgroup of patients with heart failure may not have been identified.

### Comparison with existing literature

This study concurs with the findings of Steel *et al*, who reported that out of 48 studies cited in the National Institute for Health and Care Excellence guidance on heart failure treatment, 43 (90%) were studies of uncertain relevance to patients in primary care.^14^ These findings are particularly important as there is evidence that heart failure treatments may be less effective in patients with less severe heart failure,^16^ ^,49,^ ^50^ and these drugs do account for significant morbidity.

### Implications for research and practice

The underrepresentation of patients with HFrEF and mild or absent symptoms in clinical trials has implications for GPs, who should weigh the potential benefits of initiating treatment in those with absent or mild symptoms against the risks of an adverse drug reaction. These risks are significant, although all degrees of heart failure have raised mortality and morbidity. By extrapolating data from studies of patients with more severe disease, patients and clinicians may misinterpret the potential benefits and risks. It is important that the risks and benefits are stratified by NYHA disease class.

More studies are needed using individual patient data analysis by heart failure severity, as most of the outcomes in the current studies were not reported by NYHA class. This should be complemented by observational studies using, for example, the Clinical Practice Research Datalink dataset which primarily recruit from primary rather than secondary care.
